# Florid Cystitis Glandularis with Intestinal Metaplasia in the Prostatic Urethra: a case report and review of the literature.

**DOI:** 10.1016/j.ijscr.2024.109416

**Published:** 2024-02-28

**Authors:** Oussama G. Nasrallah, Alaa Balaghi, Noura El Sayegh, Jana H. Mahdi, Sara Sinno, Rami W. Nasr

**Affiliations:** a Division of Urology, Department of Surgery, American University of Beirut Medical Center, Beirut, Lebanon; b Department of Pathology, American University of Beirut Medical Center, Beirut, Lebanon; c Faculty of Medicine, Lebanese University, Beirut, Lebanon

**Keywords:** Cystitis glandularis, Intestinal metaplasia, Florid cystitis glandularis, Prostatic urethra

## Abstract

**Introduction:**

Cystitis glandularis is a proliferative disease of the bladder epithelium usually presenting in the setting of chronic inflammation, characterized by the formation of glands in the bladder mucosa and submucosa. Intestinal metaplasia is a described process in cystitis glandularis characterized by the presence of intestinal cells and mucin production which is rare as compared to cystitis glandularis.

**Case presentation:**

We present a case of cystitis glandularis with intestinal metaplasia located in the bladder and concomitantly in the prostatic urethra. Patient underwent transurethral resection of the lesion which was unusually found in the prostatic urethra.

**Clinical discussion:**

Florid cystitis glandularis is a rare condition found in women more than in men. It usually presents with irritative lower urinary tract symptoms or hematuria which leads to its eventual diagnosis. It is usually causes by inflammation to the bladder mucosa due to infections or irritation. Patients are diagnosed through Transurethral resection of these bladder lesions found in the trigone and bladder neck region. Surgery is the standard treatment of choice. However, medical treatment may also be used to treat underlying inflammatory conditions using antibiotics, steroids, and non-steroidal anti-inflammatory agents. Radical or partial cystectomy may be performed for severe refractory cases.

**Conclusion:**

This article describes the rare occurrence of florid cystitis glandularis in the prostatic urethra and provides an overview on diagnosis, etiology, and management of the disease.

## Highlights

• Cystitis glandularis with intestinal metaplasia is a rare finding in the urinary system.
• Intestinal metaplasia may occur secondary to inflammation, or due to embryogenic remnants.
• This condition is observed more frequently in women than in men in the bladder neck and trigone area.
• Treatment options are available such as medical treatment and surgical treatment.
• Partial or radical cystectomy may be performed in severe and recurrent cases of florid cystitis glandularis.

## 1 Introduction

Cystitis glandularis is a proliferative disease of the bladder epithelium that is usually present in the setting of chronic inflammation of the bladder mucosa. It is characterized by the formation of glands in the bladder mucosa and submucosa [1]. Intestinal metaplasia is a described process in cystitis glandularis characterized by the presence of intestinal cells and mucin production which is rare as compared to cystitis glandularis. We present a case of cystitis glandularis with intestinal metaplasia located in the bladder and concomitantly in the prostatic urethra.

## 2 Methods

The work has been reported in line with the SCARE criteria [2].

## 3 Case presentation

**Fig. 1:**
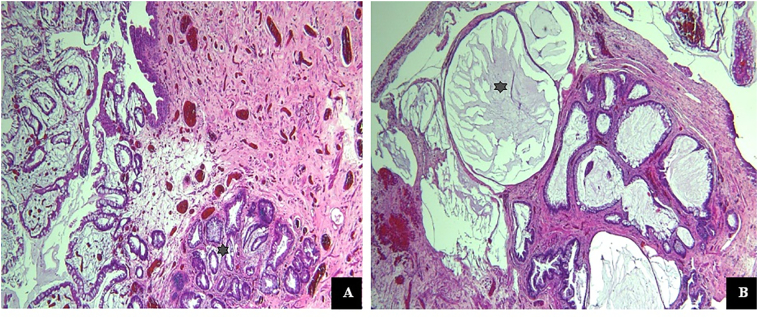
A. Cystic glandularis with intestinal metaplasia. B Mucin pooling pointed with black asterix (hematoxylin & eosin ×40).

We present a case of a 45-year-old male smoker presenting with irritative lower urinary tract symptoms, dysuria, and microscopic hematuria, that date back 22 years prior to his presentation to our institution. Back then, cystoscopy revealed bladder polyps resected in a peripheral hospital. Since then, he underwent 6 Transurethral resection of Bladder Tumors (TURBT) which would partially relieve him from the frequency, urgency, and dysuria last of which was 16 years prior to presentation. The patient had no history of recurrent Urinary tract infections, no history of radiation exposure, and no intravesical instillations. Urine analysis and culture were performed showing no urinary tract infection. Then cystoscopy was done showing polypoid lesions seen in the prostatic urethra and in the bladder neck and trigonal area. The patient underwent TURBT. Pathologic examination revealed cystitis glandularis with extensive intestinal metaplasia and mucin pooling (Fig. 1). Patient was followed up by cystoscopy every 3–6 months for monitoring of recurrence and CT scan of the Abdomen and pelvis was done on follow up which was non revealing for polyps in the bladder or upper urinary.

## 4 Discussion

Cystitis glandularis was first reported in 1761. It is a common bladder lesion distinguished by hyperplasia and metaplasia of the bladder mucosal epithelium [3]. Based on microscopic features, there are 2 types: intestinal and common. Intestinal metaplasia in urothelium can occur secondary to calculi, exstrophy, schistosomiasis, and chronic infection [4]. Incomplete intestinal metaplasia occurs when columnar epithelial metaplasia is seen in cystitis glandular associated with goblet cells. Whereas the presence of the Paneth and argentaffin cells is called complete intestinal metaplasia [5]. This case demonstrates that the urothelium lining the prostatic urethra could undergo intestinal metaplasia concurrently with the urothelium in the bladder.

The first cystitis glandularis with intestinal metaplasia was first reported in 1942. There have been 3 speculated mechanisms of pathogenesis for cystitis glandularis: 1. epithelium degenerated to an already differentiated previous stage, 2. epithelial transformation due to a pathogenic factor transforming uroepithelium to glandular epithelium, and 3. embryogenic origin theory: during bladder development, abnormal factors causes ectopic embryogenic remnant which can form glands leading to glandularis cystitis [6]. The most accepted theory is the transformation in which under certain conditions, von Bronn's nests could form and transform into glandularis cystitis.

Florid cystitis glandularis is defined as an intestinal type of cystitis glandularis in extreme differentiation [7]. It is observed more frequently in women than in men mainly in the region of the bladder neck and trigone [6], with a usual presentation of hematuria and irritative lower urinary tract symptoms as seen in our patient. Cystitis glandularis with intestinal metaplasia contains glands embedded within the intestinal epithelial cells within the lamina propria, and clusters of mucus-secreting cup-shaped cells, in addition to the commonly found columnar cells that do not contain mucin [5]. There is one report in the literature describing cystitis glandularis with intestinal metaplasia in the prostatic urethra with resection being the treatment performed as was done in our patient [8].

Diagnosis of florid cystitis glandularis of the intestinal type depends on pathology. It could be misdiagnosed with adenocarcinoma and endometrioma in women [6]. Grossly, the resected specimen looks like a polypoid mass mimicking a typical neoplasm and on histologic examination it resembles an adenocarcinoma. However, it usually lacks the architectural complexity and nuclear atypia seen in bladder cancer. This can be helpful in excluding bladder adenocarcinoma [5]. Florid cystitis glandularis and intestinal metaplasia can co-exist with bladder adenocarcinoma, but it is not a risk factor for the development of carcinoma [9]. This condition is usually treated surgically by transurethral resection and patients have a good prognosis. Yet other treatment options have been described in the literature such as partial or total cystectomy for recurrent flaccid cystitis glandularis [6,10]. Medical treatment may also be used for such cases such as the use of oral steroids, no steroidal anti-inflammatory drugs, and antibiotics [11,12]. This is a case of a rare presentation of Florid cystitis glandularis in the lower urinary system presenting usually in the bladder and a rare presentation in the prostatic urethra with one previous report in the literature making it the second case of Florid cystitis glandularis in the prostatic urethra.

## Consent and ethical approval information

Written informed consent was obtained from the patient for publication of this case report and accompanying images. A copy of the written consent is available for review by the Editor-in-Chief of this journal on request.

## Ethical approval

As this publication is a case report that contains no identifiable content to the patient, this publication was exempt from ethical approval by the Human Research Protection Program (HRPP) and its Institutional Review Board (IRB) at the American University of Beirut Medical Center, Beirut, Lebanon.

## Funding

N/A.

## Guarantor

Rami Nasr and Oussama Nasrallah.

## Research registration number

1. Name of the registry: n/a
2. Unique identifying number or registration ID: n/a
3. Hyperlink to your specific registration (must be publicly accessible and will be checked): n/a.

## CRediT authorship contribution statement

**Rami Nasr:** Conceptualization, Review & editing, Supervision
**Oussama Nasrallah:** Resources, Writing - original draft, Supervision, Review & editing
**Alaa Balaghi:** Resources, Writing - original draft, Review & editing
**Jana Mahdi:** Writing - original draft
**Noura El Sayegh:** Resources, Writing - original draft, Review & editing
**Sara Sinno:** Resources.

## Declaration of competing interest

N/A.
